# Enterovirus Infections Are Associated With the Development of Celiac Disease in a Birth Cohort Study

**DOI:** 10.3389/fimmu.2020.604529

**Published:** 2021-02-02

**Authors:** Maarit Oikarinen, Leena Puustinen, Jussi Lehtonen, Leena Hakola, Satu Simell, Jorma Toppari, Jorma Ilonen, Riitta Veijola, Suvi M. Virtanen, Mikael Knip, Heikki Hyöty

**Affiliations:** ^1^Faculty of Medicine and Health Technology, Tampere University, Tampere, Finland; ^2^Unit of Health Sciences, Faculty of Social Sciences, Tampere University, Tampere, Finland; ^3^Research, Development and Innovation Center, Tampere University Hospital, Tampere, Finland; ^4^Department of Paediatrics and Adolescent Medicine, Turku University Hospital, Turku, Finland; ^5^Institute of Biomedicine, Centre for Integrative Physiology and Pharmacology, University of Turku, Turku, Finland; ^6^Immunogenetics Laboratory, Institute of Biomedicine, University of Turku, Turku, Finland; ^7^PEDEGO Research Unit, Medical Research Centre, Department of Paediatrics, University of Oulu, Oulu, Finland; ^8^Department of Children and Adolescents, Oulu University Hospital, Oulu, Finland; ^9^Department of Public Health Solutions, Finnish Institute for Health and Welfare, Helsinki, Finland; ^10^Center for Child Health Research, Tampere University and Tampere University Hospital, Tampere, Finland; ^11^Pediatric Research Center, Children’s Hospital, University of Helsinki and Helsinki University Hospital, Helsinki, Finland; ^12^Research Program for Clinical and Molecular Metabolism, Faculty of Medicine, University of Helsinki, Helsinki, Finland; ^13^Fimlab Laboratories, Pirkanmaa Hospital District, Tampere, Finland

**Keywords:** enterovirus, celiac disease, tissue transglutaminase autoantibodies, Finnish Diabetes Prediction and Prevention study, enzyme immunoassay (EIA), conditional logistic regression

## Abstract

Enterovirus and adenovirus infections have been linked to the development of celiac disease. We evaluated this association in children who developed biopsy-proven celiac disease (N = 41) during prospective observation starting from birth, and in control children (N = 53) matched for the calendar time of birth, sex, and HLA-DQ genotype. Enterovirus and adenovirus infections were diagnosed by seroconversions in virus antibodies in longitudinally collected sera using EIA. Enterovirus infections were more frequent in case children before the appearance of celiac disease-associated tissue transglutaminase autoantibodies compared to the corresponding period in control children (OR 6.3, 95% CI 1.8–22.3; p = 0.005). No difference was observed in the frequency of adenovirus infections. The findings suggest that enterovirus infections may contribute to the process leading to celiac disease.

## Introduction

Celiac disease is one of the most common immune-mediated diseases with a prevalence of 0.5–1% in the general population. The prevalence is rapidly increasing, particularly in Western countries, suggesting that, in addition to the well-known genetic susceptibility (especially HLA-DQ2 and HLA-DQ8 risk genes), environmental factors play an important role in the pathogenesis. The autoimmune process targets the tissue transglutaminase (tTG) and gluten is the well-known trigger. However, the majority of gluten-exposed and genetically susceptible individuals will not develop the disease, suggesting that other environmental factors may be involved in the pathogenesis (1).

Several studies have suggested that certain virus infections, particularly those caused by enteral viruses, are associated with the development of celiac disease (2). Gastroenteritis (3) and certain specific enteric viruses, including rotavirus (4), adenovirus (5), and reovirus (6), have been associated with celiac disease. A recent prospective study showed association between parechoviruses and celiac disease (7). Recently, enterovirus infections were linked to the initiation of the disease process in two prospective birth cohort studies while other tested viruses did not (8, 9) supporting a previous study showing enterovirus RNA and protein in the small intestinal biopsy samples collected from patients with celiac disease (10). On the other hand, one recent prospective study showed no association between enterovirus or any other studied viruses and celiac disease (11).

The mechanisms of these associations have remained unclear. One hypothesis suggests that viral infections are involved in immune activation and breakdown of tolerance against gluten in genetically susceptible individuals. Viral infections early in life could affect mucosal immune system maturation and cause also long-term changes in the commensal microbiota (12).

The aim of this study was to confirm the association that has recently been observed between enterovirus infections and later development of celiac disease in two prospective birth cohort studies. The current study is based on a third independent prospective birth cohort. In contrast to these previous studies in which infections were diagnosed by detecting viral genomes from serial stool samples we employed virus serology to detect increases in enterovirus antibody levels between consecutive follow-up serum samples. In parallel, adenovirus infections were diagnosed using the same approach.

## Materials and Methods

The study included altogether 41 children who had developed celiac disease-associated tissue transglutaminase autoantibodies (tTGA) and whose celiac disease diagnosis had been confirmed by morphological examination of duodenal biopsy sample (ESPGHAN criteria) (13–15). Also, 53 control children were included: one control child for 29 and two control children for 12 case children. Each case-control pair was pairwise matched for calendar month and year of birth (+/- 2 months), sex, city of residency and HLA-DQ alleles. The children participated in the prospective, observational Finnish Diabetes Prediction and Prevention (DIPP) study in which children genetically at risk to develop type 1 diabetes (T1D) and celiac disease are followed from birth (16). Blood samples taken at 3–12 months’ intervals were systematically screened for tTGA as previously described (13–15).

Virus antibodies were analyzed from all follow-up serum samples collected from case children from birth until the appearance of tTGA, including the first serum sample taken after tTGA seroconversion (mean 12 samples per child). A small intestinal biopsy sample was taken after seroconversion to positivity for tTGA at the mean age of 5 years (range 2–14 years) from all case children to confirm the diagnosis of celiac disease. The corresponding samples were analyzed from the tTGA negative control children (mean 10 samples per child).

Serum IgA antibodies against tTGA were measured using a recombinant human TGA kit (Celikey; Pharmacia Diagnostics, Freiburg, Germany) with values of 5–8 U/ml regarded as equivocal and >8 U/ml as positive, as suggested by the manufacturer (13).

IgG class antibodies against enterovirus and adenovirus were analyzed from follow-up serum samples using EIA as earlier described (17). Briefly, sucrose gradient purified coxsackievirus B1 (produced in house), and adenovirus type 2 hexon protein (SERION Immunologics, Wurzburg, Germany) were used as antigens to coat microtiter plates in 1 µg/ml concentration. Serum samples were tested in 1/1,000 dilution and a two-fold increase in the antibody levels (absorbance values) between sequential samples was used as a criterion for a virus infection.

Information on breastfeeding as well as the first introduction to gluten were provided by the parents of the children using structured dietary questionnaires (18). Breastfeeding was analyzed as a duration of both exclusive breastfeeding and total breastfeeding. The amount of gluten intake was based on 3-day food records (19, 20). Median age at gluten introduction was 5.0 (IQR 5.0–6.0) months and mean gluten intake at age of 6 months was 0.38 (SD 0.60) grams/day. This information about breastfeeding and gluten intake was available from 34 case children and 44 control children.

**Figure 1:**
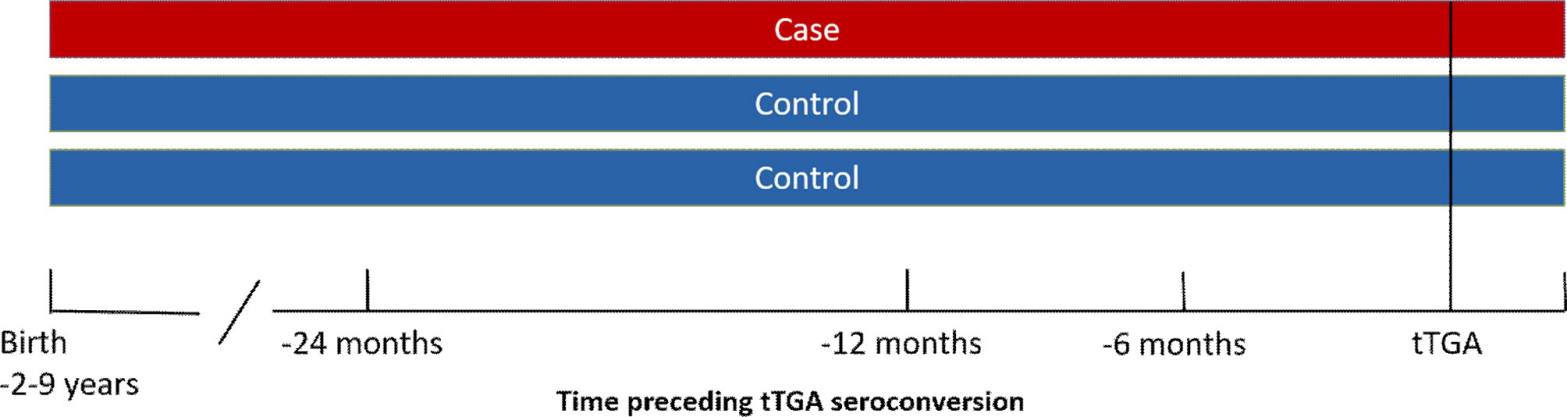
Study design. Serum samples from a case child and one or two control children were analyzed for enterovirus and adenovirus IgG antibodies in different time windows preceding the appearance of tissue transglutaminase autoantibodies.

Statistical analyses were performed in R 4.0.2 software using conditional logistic regression which takes into account those case-control pairs which were discordant for the infection. Time windows of 6, 12, and 24 months prior to the appearance of tTG autoantibodies were used to compare the occurrence of enterovirus and adenovirus infections in case and control children. Duration of breastfeeding and age at the first introduction and amount of gluten intake were tested as possible confounding factors. The study design is shown in **Figure 1**.

## Results

The mean age of tTGA seroconversion in the case children was 5 years (range 2–9 years). Enterovirus infections were frequent in both case and control children as altogether 98% of case children and 96% of control children had had at least one infection by the time of tTGA seroconversion. Adenovirus infections were also common (90% of case children and 81% of control children had had at least one infection).

**Figure 2:**
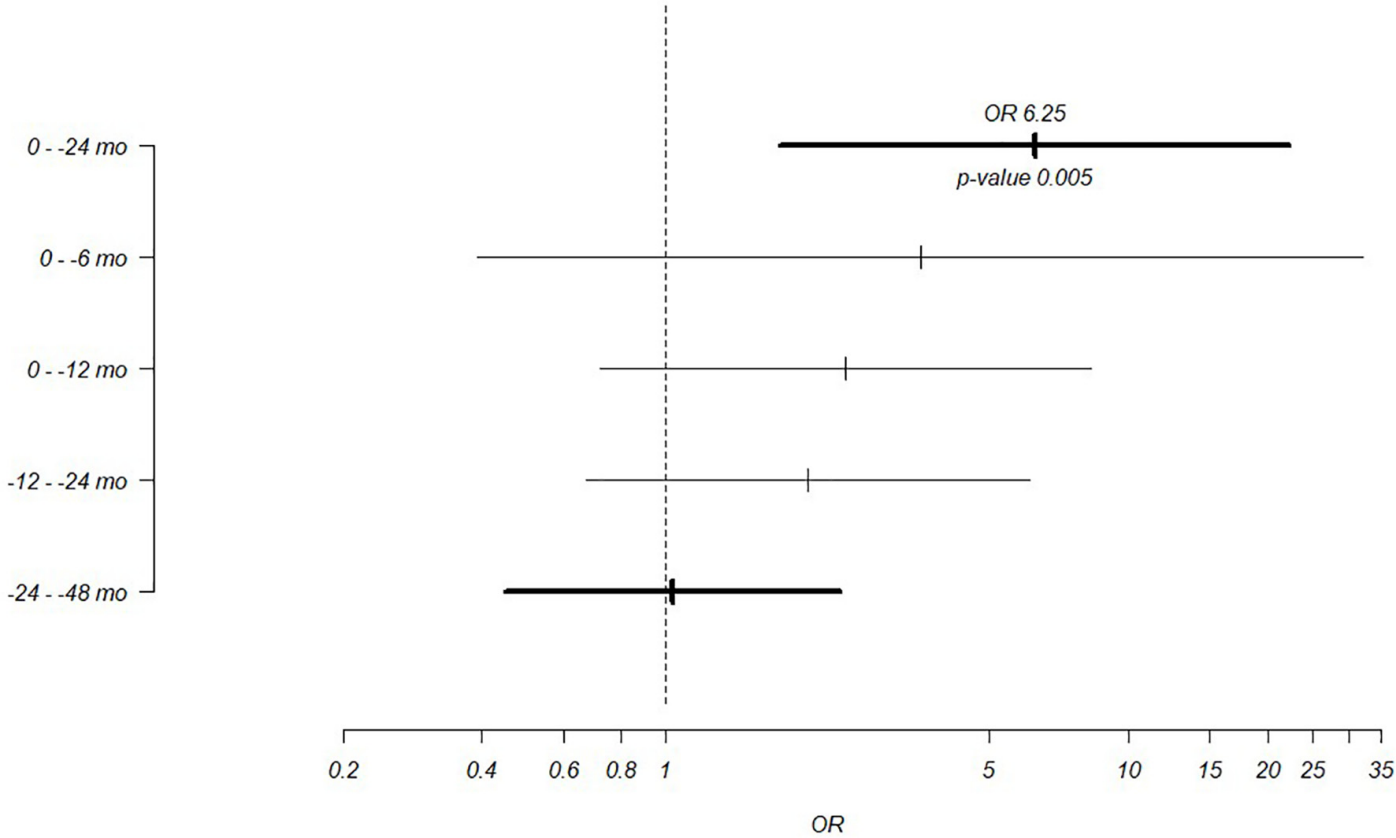
Odds ratios for the association between enterovirus infections and the appearance of tissue transglutaminase autoantibodies in different time windows. Broader time windows of 0–24 months and 24–48 months preceding the appearance of autoantibodies are marked with bolded lines.

Time-dependent analyses showed that case children had significantly more enterovirus infections than control children during the 24 months preceding tTGA seroconversion: 51% of case children compared to 25% of control children had enterovirus infection during this time period (OR = 6.26, 95% CI = 1.76–22.28, p = 0.005; **Table 1**; **Figure 2**). A similar but statistically non-significant trend was seen in infections during the 6 and 12 months preceding tTGA seroconversion (**Table 1**; **Figure 2**). Adjustment for breastfeeding (duration of both exclusive breastfeeding and total breastfeeding) or gluten intake (grams/day at the age of 6 months) did not have any effect on the association between enterovirus infections and tTGA seroconversion (adjustment for exclusive breastfeeding: OR = 4.45, 95% CI = 1.15–17.27, p = 0.031; adjustment for total breastfeeding: OR = 4.3, 95% CI = 1.12–16.44, p = 0.033; adjustment for gluten intake: OR = 5.58, 95% CI = 1.18–26.52, p = 0.031).

**Table 1:** Proportion of case and control children with enterovirus and adenovirus infections in different time windows preceding the appearance of tissue transglutaminase autoantibodies.

	0–6 months prior to tTGA+ (N = 72)			0–12 months prior to tTGA+ (N = 100)			0–24 months prior to tTGA+ (N = 109)		
	Cases (N = 36)	Controls (N = 36)	OR (95% CI), p value	Cases (N = 41)	Controls (N = 59)	OR (95% CI), p value	Cases (N = 41)	Controls (N = 68)	OR (95% CI), p value
Enterovirus	22%	11%	3.56 (0.39,32.19), p = 0.258	29%	15%	2.45 (0.72,8.3), p = 0.151	51%	25%	6.26 (1.76,22.28), p = 0.005
Adenovirus	33%	28%	0.95 (0.33,2.78), p = 0.928	37%	31%	1.23 (0.52,2.89), p = 0.640	54%	46%	1.52 (0.67,3.46), p = 0.314

In contrast, the frequency of adenovirus infections did not differ between case and control children in any time period preceding tTGA seroconversion (**Table 1**). Adjustment for breastfeeding (duration of both exclusive breastfeeding and total breastfeeding) or gluten intake (grams/day at the age of 6 months) did not have any effect on this association (data not shown).

No difference was seen between case and control children in enterovirus or adenovirus infections that occurred after tTGA seroconversion or earlier in life (**Figure 2**) before the 24 months preceding the seroconversion nor when cases and controls were compared among boys and girls separately in any of the time periods (data not shown).

## Discussion

This is the largest study carried out among prospectively followed children to study the association between virus infections and celiac disease. The results confirm the findings recently reported from two case-control series nested within two smaller birth cohort studies (8, 9). The facts that the present study, together with the two previous prospective studies find such an association even though they were carried out in different populations and used different technologies to diagnose enterovirus infections (serology vs. detection of viral nucleic acids by RT-PCR or next generation virome sequencing in stool samples) suggest that enterovirus infections may indeed be linked to the development of celiac disease.

The mechanisms of this association are not known. Theoretically, enterovirus infections may interact with gluten in the intestinal mucosa and create an inflammatory environment which activates antigen presenting cells thus augmenting immune responses against gluten and deaminated gluten peptides. The fact that gut mucosa and mucosal immune system are the primary replication sites of enteroviruses fits with this hypothesis, particularly since the virus can replicate in intestinal tissues for quite a long time, even for several weeks. In fact, in the two previous studies the association between enterovirus infections and celiac disease autoimmunity (CDA) was linked to infections that occurred after the age of one year, i.e., after the introduction of gluten (8, 9). In one of them an interaction with cumulative amount of ingested gluten was found (8). In our study, the association was seen in enterovirus infections that occurred within 24 months before CDA seroconversion, thus covering the time after gluten introduction, and adjustment for gluten intake did not affect the association between enterovirus infections and CDA.

In addition, molecular mimicry between enterovirus proteins and gliadin/tTG could induce cross-reactive immune responses and autoimmunity. However, no linear mimicry epitopes have been reported in enteroviruses and gliadin or tTG. In addition, the type of the enterovirus causing the infection could also be an important factor in the pathogenesis. In the present study, it was not possible to identify the type of enteroviruses causing these infections since the used antibody assay cannot distinguish different enterovirus types from each other. In the previous studies, the Norwegian cohort indicated a risk association for both species A and B enteroviruses with higher odds ratio for species B than species A enteroviruses (9), while the international TEDDY cohort showed risk association only for species B enteroviruses (particularly group B coxsackieviruses) (8). However, both studies lacked statistical power to analyze the associations of individual virus types with celiac disease. Thus, further studies with larger numbers of outcomes are needed to find out whether the enterovirus-celiac disease association links to certain specific enterovirus types.

One of the strongest assets of the present study is based on its design; a case-control study nested in a prospective birth cohort. This design made it possible to study the time-relationships between infections and the appearance of tTGA and celiac disease. The observed time-order showing that the excess of enterovirus infections occurred before tTGA positivity and diagnosis of celiac disease suggests that infections may indeed trigger celiac disease and not *vice versa*. The diagnosis of celiac disease was confirmed by intestinal biopsy making the study endpoint reliable and clinically relevant. An additional advantage is that the used serological assays were able to detect infections that have occurred between the study visits since IgG class enterovirus antibody responses remain elevated long after the infection. This is in contrast to direct virus detection by RT-PCR which requires the presence of the virus in the sample, leading to under-diagnosis of infections in this kind of cohort studies where samples are collected according to preset regular intervals.

The study also has limitations which need to be taken into account when interpreting the data. Most importantly, it cannot define whether the observed associations are causal or whether they could be caused by an interference of some unknown confounding factors. Careful matching of case and control children eliminated several possible confounding factors that might be associated with the season or year of birth, sex, HLA-DQ genotype or geographic region, but still we cannot exclude the possibility that some other factors could have biased the findings. However, no difference was seen in adenovirus infections between cases and controls, suggesting that there was no general difference in viral exposures between the case and control subjects. Another important limitation is that the study covered only young children and one country. However, similar findings from the previous studies covering several other populations (8, 9) indicate that the association is not country-specific.

It should also be noted that based on the current data it is challenging to estimate how large proportion of celiac disease could be related to enterovirus infections. Theoretically, this proportion could be relatively high since as many as 51% of the patients had had infection during the 2 years preceding tTGA seroconversion, compared to 25% of the controls. According to these figures and the OR of 6.3 the population attributable risk per cent (PAR%) would be 57%. Thus, in case this association would be causal, enterovirus infections could contribute to the development of more than half of the celiac disease cases. However, intervention studies, such as vaccination trials, would be needed to find out whether the association is causal and how large a proportion of cases could be attributable to enterovirus infections. Vaccines against certain non-polio enteroviruses such as enterovirus 71 (21) or group B coxsackieviruses (22) have already been licensed or are in clinical development, which may open possibilities to carry out intervention trials in the future.

In conclusion, the present study supports previous observations showing an association between enterovirus infections and celiac disease. Further studies are needed to evaluate possible mechanisms behind this association and to find out whether these observations could offer an option to prevent celiac disease by vaccines or other antiviral strategies.

## Data Availability Statement

The raw data supporting the conclusions of this article will be made available by the authors, without undue reservation.

## Ethics Statement

The study was approved by the ethics committees of the participating university hospitals (Tampere University Hospital, Turku University Hospital and Oulu University Hospital) and the parents of the participating children gave their informed written consent to the participation in the study. Written informed consent to participate in this study was provided by the participants’ legal guardian/next of kin.

## Author Contributions

MO designed the laboratory analyses, analyzed and interpreted the data, and prepared the manuscript. LP designed the laboratory analyses, and analyzed and interpreted the data. JL performed the statistical analyses. LH and SV provided the nutritional data. SS, JT, JI, RV, MK, and HH provided the clinical data and samples. HH designed the study and helped prepare the manuscript. All co-authors reviewed the manuscript. MO is the guarantor of this work. All authors contributed to the article and approved the submitted version.

## Funding

This study was supported by grants from the Finnish Technology Agency TEKES (currently Business Finland), Academy of Finland (276475, 308066), Reino Lahtikari Foundation, Sigrid Juselius Foundation and the Competitive State Research Financing of the Expert Responsibility area of Tampere, Turku and Oulu University Hospitals [X50040, 9E082, 9F089, 9G087, 9H092, 9J147, 9K149, 9L042, 9L117, 9M036, 9M114, 9N086, 9P057, 9R055, 9S074, 9T072]. The study sponsors had no role in the design, analysis or writing of this article.

## Conflict of Interest

HH and MK are shareholders and members of the board of Vactech Ltd. which develops vaccines against picornaviruses.

The remaining authors declare that the research was conducted in the absence of any commercial or financial relationships that could be construed as a potential conflict of interest.
